# Which adults in the Paris metropolitan area have never been tested for HIV? A 2010 multilevel, cross-sectional, population-based study

**DOI:** 10.1186/s12879-015-1006-9

**Published:** 2015-07-22

**Authors:** Véronique Massari, Annabelle Lapostolle, Marie-Catherine Grupposo, Rosemary Dray-Spira, Dominique Costagliola, Pierre Chauvin

**Affiliations:** Sorbonne Universités, UPMC Univ Paris 06, INSERM, Institut Pierre Louis Institute d’Epidémiologie et de Santé Publique (IPLESP UMRS 1136), Department of Social Epidemiology, F-75012 Paris, France; Sorbonne Universités, UPMC Univ Paris 06, INSERM, Institut Pierre Louis Institute d’Epidémiologie et de Santé Publique (IPLESP UMRS 1136), Department of HIV Clinical Research, F-75013 Paris, France

**Keywords:** HIV, Sexual behavior, Prevention, Gender, Multilevel, Cross-sectional studies, Social inequalities, Immigration status, Neighborhood

## Abstract

**Background:**

Despite the widespread offer of free HIV testing in France, the proportion of people who have never been tested remains high. The objective of this study was to identify, in men and women separately, the various factors independently associated with no lifetime HIV testing.

**Methods:**

We used multilevel logistic regression models on data from the SIRS cohort, which included 3006 French-speaking adults as a representative sample of the adult population in the Paris metropolitan area in 2010. The lifetime absence of any HIV testing was studied in relation to individual demographic and socioeconomic factors, psychosocial characteristics, sexual biographies, HIV prevention behaviors, attitudes towards people living with HIV/AIDS (PLWHA), and certain neighborhood characteristics.

**Results:**

In 2010, in the Paris area, men were less likely to have been tested for HIV at least once during their lifetime than women. In multivariate analysis, in both sexes, never having been tested was significantly associated with an age younger or older than the middle-age group (30–44 years), a low education level, a low self-perception of HIV risk, not knowing any PLWHA, a low lifetime number of couple relationships, and the absence of any history of STIs. In women, other associated factors were not having a child <20 years of age, not having additional health insurance, having had no or only one sexual partner in the previous 5 years, living in a cohabiting couple or having no relationship at the time of the survey, and a feeling of belonging to a community. Men with specific health insurance for low-income individuals were less likely to have never been tested, and those with a high stigma score towards PLWHA were more likely to be never-testers.

Our study also found neighborhood differences in the likelihood of men never having been tested, which was, at least partially, explained by the neighborhood proportion of immigrants. In contrast, in women, no contextual variable was significantly associated with never-testing for HIV after adjustment for individual characteristics.

**Conclusions:**

Studies such as this one can help target people who have never been tested in the context of recommendations for universal HIV screening in primary care.

## Background

In high-income countries, publications on the barriers to and/or the facilitators of HIV testing remain scarce [1, 2], especially those focusing on the situation of their immigrant populations [3–7]. In France, because of the legal restrictions on collecting and processing data on ethnicity, religion and immigration status, there are few studies on the social and migrational determinants of HIV testing [8–10].

Despite universal access to HIV screening and treatment and one of the highest annual HIV testing rates in Europe (103 per 1,000 inhabitants) [11], the Paris region is characterized by a high proportion of HIV-positive tests (4.5 per 1,000 tests) and a large number of people who are unaware of their HIV infection (estimated at around 13,000 in a regional population of 11.9 million people) [12, 13]. In this context, new screening strategies have been considered, which consist not only in promoting regular testing of high-risk individuals, but also in increasing the uptake of HIV screening by people who have never been tested. Indeed, in the past 5 years, the percentage of never-testers has decreased significantly but is still significant in this region. In 2010, a regional KABP survey found that 33.9 % of men and 21.5 % of women aged 18–54 years had never been tested during their lifetime (these proportions were 47.0 % and 33.6 % in 2004, respectively) [11]. The regional incidence of AIDS is estimated to be approximately 1,500 cases each year. In 2010, 60 % of them were unaware of their HIV status at the time of diagnosis, and 40 % had never been tested [14].

Our objectives were to collect certain socioeconomic status indicators, origin, sexual biographies, attitudes and behaviors regarding HIV testing and prevention, and neighborhood characteristics, in a representative sample of the adult, French-speaking population in the Paris metropolitan area and to determine those associated with no lifetime uptake of HIV testing.

## Methods

The SIRS (a French acronym for health, inequalities and social ruptures) cohort is the first large, representative, population-based cohort created to study the social determinants of health and health-care utilization in the field of social epidemiology in France [15, 16]. In 2005, at inclusion, our study population was a multistage random sample of the adult French-speaking population living in the Paris metropolitan area (also called “Greater Paris”, consisting of the City of Paris and its three adjacent *départements*, which constitute the core area of the entire Paris region, with 6.6 million inhabitants). The primary sampling units were census blocks including about 2000 inhabitants each. Fifty of them were randomly selected from the 2595 eligible census blocks according to their socioeconomic type. Subsequently, 60 households in each census block were chosen at random. Lastly, one adult in each household was randomly selected by the next-birthday method and interviewed at home.

In 2010, 47.2 % of the 3006 participants included in 2005 were reinterviewed (2.6 % were deceased, 1.8 % were too sick to answer our questions, 13.9 % had moved out of the 50 surveyed IRISs, 2.7 % were absent during the survey period, 18.4 % declined to participate, and 13.4 % were lost to follow-up). Their sex ratio and mean age were similar to those of the individuals who were not reinterviewed. The individuals lost to follow-up were younger and better off than the others, but neither their health status nor the socioeconomic type of their census block of residence was different. Those absent during the survey period had a lower socioeconomic status and were mostly immigrants. In each census block, the individuals who were not reinterviewed in 2010 were replaced by means of a random procedure similar to the one used in 2005, up to a final sample size of 60 adults interviewed per census block. The refusal rate among the newly contacted individuals was 29 % (the same as in 2005).

In this paper, data collected in 2010 were examined cross-sectionally. The independent variables were selected from the SIRS dataset for the relevance of their association with HIV testing [17] or, more generally, with preventive health-care activities [18]. The analyses were limited to the 2621 subjects under 70 years of age to avoid a possible memory bias in the older individuals and to compare our data with those from other French studies.

### Ethics

This cohort study had received legal authorization from two national authorities for non-biomedical research [19]: the *Comité consultatif sur le traitement de l’information en matière de recherche dans le domaine de la santé* (CCTIRS) (authorization number 904251) and the *Commission nationale de l’informatique et des libertés* (CNIL) (authorization number 05-1024). The participants provide their verbal informed consent. Written consent was not necessary because this survey did not fall into the category of biomedical research (as defined by French law) and did not collect any personal identification data.

### Measures

#### Outcome

The outcome variable was no lifetime HIV testing.

#### Demographics

A first set of independent variables examined in this study consisted of demographics, which included age and ‘immigration status’. The latter was defined and categorized on the basis of both the individual’s and his/her parents’ nationality (at the time of the survey or of their death), distinguishing between French, born to two French parents; French, born to at least one foreign parent; and foreigners. Because HIV screening for all pregnant women had been widely offered since 1990 in France [20], having a child <20 years of age (or being pregnant at the time of the survey) was also considered.

#### Socioeconomic status

(SES)**-** Several characteristics related to the participants’ SES were taken into account: education level (none or primary school, secondary school, and postsecondary), employment status (actively employed vs. all others, including unemployed, retired, student and inactive), occupational category (coded as never having worked, blue-collar, tradespeople/salespeople/shopkeepers and intermediate occupation, lower white-collar, and upper white-collar), and monthly income per consumption unit (CU), as calculated according to the OECD scale [21].

#### Health care utilization status

This dimension was explored by examining two variables: health insurance status and having or not having a regular general practitioner.

#### HIV risk and stigma

The participants’ self-perception of HIV risk (ranked as low or high) and their knowing or not knowing people living with HIV/AIDS were taken into account. Five questions regarding attitudes towards PLWHA (namely, would accept to 1) work with them, 2) have a meal at their home, 3) go on vacation with them, 4) have protected sex with them, and 5) let them look after their children or grandchildren) were combined to calculate a stigma score, which ranged from 0 and 10 and increased with the level of negative attitude towards PLWHA. For each gender, this score was dichotomized into two categories (≤ or > 2, i.e., the mean score value for the entire population and also by gender).

#### Sexual biographies, attitudes and behaviors

The following variables were taken into account: self-reported sexual orientation (homosexual or bisexual, heterosexual, and not answered), the number of sexual partners of each sex, the number of sexual partnerships during the previous 5 years (one partnership, multiple partnerships or no partnerships), couple status at the time of the survey (no relationship, love affair, non-cohabiting couple or cohabiting couple), and the lifetime number of couple relationships. The interviewees were also asked about their STI history (with a single question: “Have you ever had an STI?”, with no further details).

#### Social integration

Certain characteristics pertaining to the participants’ social ties and social support were taken into consideration as well: a feeling of being supported (or of not being supported) by friends, relatives or neighbors (“social support” in the rest of this paper), living (or not) living alone, a sense of belonging to a community, and their religious affiliation and practice, if any (but not their religion per se, as this would not have been very acceptable in France).

#### Neighborhood characteristics

At the neighborhood level, four socioeconomic characteristics of the census blocks of residence were considered. The mean monthly household income was calculated using the 2007 income tax database (provided by French tax authorities) and divided into quartiles. The proportions of immigrants, unemployed residents, and people with a low education level (no education or primary school) were available in the 2007 population census data provided by the French National Institute of Statistics and Economic Studies.

### Statistical analyses

The differences in characteristics among the participants who reported having been tested at least once for HIV and those who had not were investigated using χ^2^ analysis or the Fisher exact test. Since sexual biographies, attitudes and behaviors vary according to gender [22, 23], all the analyses were stratified by sex. All of the univariate statistical analyses were weighted to take into account the sampling method and the poststratification adjustment for age and gender according to the 2007 general population census data [16]. A two-sided *p*-value <0.05 was considered statistically significant.

To build the final model for each sex, we used the following modeling strategy. First, a null model was computed to estimate the area-level variations without any covariables. Second, we considered three groups of independent variables (1. demographics and SES; 2. the other individual variables, i.e., those concerning HIV risk and stigma toward PLWHA, sexual biographies, attitudes and behaviors, and social integration; and 3. the neighborhood variables) and performed a preselection of the independent variables associated with our outcome in each of these groups. To do this, all the variables associated with our dependant variable with a *p*-value <0.20 in univariate analysis (Table 1) were selected manually (using a backward-selection procedure based on the Hosmer & Lemeshow approach [24] with a threshold *p* < 0.10) and checking for multicollinearity. Third, in Model 1, only the preselected demographic and socioeconomic variables were included and further selected using the same procedure but with a threshold *p* < 0.05. Fourth, in Model 2, the other individual variables previously preselected were added to Model 1 and backward-selected with a threshold *p* < 0.05. Finally, all the contextual variables were added to Model 2 and selected in Model 3 in the same manner. Since no neighbourhood variable was found to be significantly associated in women, the final model was Model 2 (Table 3).

**Table 1:** Comparison of the characteristics of the HIV-tested and never-tested men (*n* = 1261) and women (*n* = 1360) in 2010

	Overall (*n* = 2621)	%	Never-tested men (*n* = 537)	%	*P*-value (tested vs. never-tested)	Never-tested women (*n* = 451)	%	*P*-value (tested vs. never-tested)
Age								
18–29 years	655	25.0	162/332	48.7	<.0001	133/323	41.3	<.0001
30–44 years	959	36.6	144/455	31.6		78/504	15.5	
45–59 years	700	26.7	143/332	43.0		140/367	38.1	
60–69 years	308	11.7	89/142	63.0		99/166	59.8	
Immigration status								
French/French parents	1688	64.4	329/832	39.5	.0031	257/856	30.0	.001
French/foreign parent (s)	544	20.7	116/243	48.0		111/301	36.7	
Foreigners	389	14.8	92/187	49.4		83/203	40.9	
Children and pregnancy								
No child under 20 years of age	1921	73.3	514/1201	42.8	.5360	333/720	46.3	<.0001
Child under 20 years of age^a^	701	26.7	23/60	38.8		118/640	18.4	
Education level								
None or primary school	145	5.6	52/75	68.8	<.0001	50/70	71.3	<.0001
Secondary school	941	35.9	234/468	50.1		173/473	36.6	
Postsecondary	1535	58.4	251/718	35.0		228/817	27.9	
Employment status								
Actively employed	1940	74.0	386/1012	38.1	<.0001	233/928	25.1	<.0001
All others	682	26.0	151/249	60.7		218/432	50.4	
Socio-occupational category								
Never worked	249	9.5	63/83	76.4	<.0001	91/166	54.9	<.0001
Blue collar	190	7.2	88/162	54.0		13/27	48.8	
Lower white-collar	948	36.2	147/362	40.7		202/586	34.5	
Tradespeople, salespeople	479	18.1	93/238	39.2		74/237	22.2	
Upper white-collar	758	28.9	146/416	35.1		70/342	20.5	
Monthly income (€/CU)								
First quartile (< 1100)	652	24.9	150/305	49.2	.0002	143/347	41.4	.0010
Second quartile (1100–1700)	663	25.3	142/304	46.8		113/359	31.5	
Third quartile (1701–2500)	657	25.1	130/333	39.0		84/324	27.3	
Fourth quartile (≥2501)	649	24.8	115/319	36.1		106/330	32.1	
Health insurance status	final							
HI + additional insurance	2104	80.4	416/976	42.6	0.0380	345/1128	30.6	<0.0001
HI for the poor	178	6.8	27/86	31.4		36/92	39.1	
HI with no additional insurance	335	12.8	94/198	47.5		66/137	48.2	
Self-perception of HIV risk								
High	708	27.0	73/349	20.9	<.0001	49/359	13.7	<.0001
Low	1913	73.0	465/912	51.0		402/1001	40.1	
Knowing PLWHA								
Yes	1650	63.0	396/807	49.1	<.0001	341/844	40.4	<.0001
No	971	37.0	141/455	31.1		110/516	21.3	
Stigma towards PLWHA								
Score ≤ 2	1740	66.4	320/840	38.1	<.0001	267/900	29.7	.0002
Score > 2	882	33.6	217/421	51.6		184/460	39.9	
Self-reported sexual orientation								
Heterosexual	2515	95.9	522/1190	43.9	<.0001	435/1325	32.9	<.0001
Homo/bisexual	75	2.9	6/58	10.2		1/16	9.4	
Not answered	31	1.2	10/13	76.9		14/19	74.9	
Sexual partnerships during the previous 5 years								
No partnerships	243	9.3	43/84	51.9	<.0001	99/159	61.9	<.0001
One partnership	1638	62.5	336/712	47.2		302/926	32.6	
Multiple partnerships	740	28.2	158/465	33.9		50/274	18.1	
Couple status at the time of the survey								
Not in a relationship	569	21.7	143/279	51.5	<.0001	132/290	45.7	<.0001
Love affair	335	12.8	50/157	32.0		59/178	32.9	
Non-cohabiting couple	156	5.9	21/82	25.5		15/73	20.3	
Cohabiting couple	1562	59.6	323/743	43.4		245/818	29.9	
Lifetime number of couple relationships								
None	556	21.2	160/320	50.0	<.0001	116/237	49.0	<.0001
One	1317	50.3	285/565	50.5		268/753	35.6	
≥ 2	747	28.5	92/377	24.5		67/370	18.0	
History of STI								
No	2260	86.2	513/1113	46.1	<.0001	409/1147	35.7	<.0001
Yes	361	13.8	24/148	16.4		42/213	19.5	
Feeling of being supported								
Yes	2597	99.1	533/1248	42.7	0.561	443/1349	32.8	0.0082
No	24.5	0.9	5/14	35.7		8/11	72.7	
Living alone								
Yes	414	15.8	75/223	33.6	0.002	73/190	38.4	0.1077
No	2207	84.2	463/1038	44.6		378/1169	32.3	
Sense of belonging to a community								
No	1806	68.9	347/872	39.8	.0024	273/934	29.3	<.0001
Yes	815	31.1	191/390	48.9		177/426	41.7	
Practice of a religion								
Practices a religion regularly	492	18.8	86/183	47.1	.1313	136/309	44.0	<.0001
Practices a religion but not on a regular basis	483	18.4	85/199	42.8		97/284	34.3	
Affiliation but does not practice	699	26.7	154/353	43.6		101/346	29.1	
No practicing or affiliation	949	36.2	212/526	40.4		117/422	27.8	
Neighborhood variables								
Mean annual household income (€/CU)								
First quartile (< 17,000)	392	14.9	107/182	58.4	<.0001	81/209	38.8	.0026
Second quartile (17,000–21,999)	673	25.7	146/330	44.2		133/343	38.9	
Third quartile (22,000–29,519)	776	29.6	157/375	41.8		112/401	28.0	
Fourth quartile (≥ 29,520)	780	29.8	128/373	34.3		124/406	30.4	
Proportion of immigrants								
First quartile	791	30.2	155/378	41.1	.0001	113/413	27.3	.0011
Second quartile	735	28.0	127/385	33.1		113/350	32.3	
Third quartile	585	22.3	111/257	43.3		124/327	38.0	
Fourth quartile	511	19.5	143/241	59.4		101/269	37.4	
Proportion of unemployed persons								
First quartile	707	27.0	130/334	39.0	.0003	112/373	29.9	.0008
Second quartile	835	31.9	156/399	39.2		134/436	30.7	
Third quartile	622	23.7	143/315	45.5		98/306	32.0	
Fourth quartile	457	17.4	107/213	50.3		107/244	44.0	
Proportion of persons with a primary education or less								
First quartile	868	33.1	153/417	36.7	.0001	115/451	25.4	.0001
Second quartile	831	31.7	162/406	39.9		141/425	33.2	
Third quartile	515	19.7	112/243	46.3		110/272	40.3	
Fourth quartile	406	15.5	110/195	56.2		85/211	40.3	

^a^ including 42 pregnant women

For each characteristic, the category with the lowest number of never-testers was used as the reference in the multivariate models. All the analyses used STATA software (STATA® v.12; STATA College Station, TX) with the xtmelogit procedure (specifying that collected data were clustered by census block). At each step, the level 2 variance was estimated to test for area-level variation, and the median odds ratio (MOR) was calculated. The MOR measures the median value of the adjusted OR between the most and least at-risk individual when comparing all pairs of neighborhoods [25].

## Results

### Characteristics of the study population

The sample consisted of 1261 men (48.1 %) and 1360 women (51.9 %). The participants’ mean age (± SD) at the time of the interview was 41.6 (±15.4) years for the men and 42.3 (± 13.1) years for the women. With regard to SES (Table 1), 58.4 % had a higher education level, 74.0 % were actively employed, 21.7 % were not in a couple relationship at the time of the survey, and 15.8 % were living alone. A sense of belonging to a community was shared by 31.1 % of the study population, and 37.2 % practiced a religion.

In 2010, 42.6 % (*n* = 537) of the men and 33.2 % (*n* = 451) of the women reported that they had never been tested for HIV during their lifetime. The men were more likely to have never been tested for HIV than the women (42.6 % vs. 33.2 %, *p* < 0.001). Of the 1639 (62.0 %) participants tested at least once in their lifetime, 56.8 % had been tested at least once at their request, 56.8 % during a systematic or routine examination (including pregnancy, marriage, blood donations and preop exams), and 20 % at their physician’s request. Of the participants who had been tested, 39.7 % had been so during the previous 24 months. The main reasons given for no testing were low risk perception (93 %), fear of HIV disease (3 %) and fear of HIV diagnosis disclosure (2 %).

The proportion of ever-testers varied greatly according to their neighborhood of residence, from 7.7 % (95 % CI: 0.0–19.6) to 85.7 % (95 % CI: 70.7–100.0) for the men, and from 19.3 % (95 % CI: 2.3–36.3) to 90.7 % (95 % CI: 80.1–100.0) for the women.

### Factors associated with no lifetime HIV testing in men

In the men (Table 1), the factors associated with never-testing for HIV in univariate analysis were a young (<29 years) or an old age (>60 years), being a foreigner or a French person born to at least one foreign parent, a lower education level, not being actively employed, being a blue-collar worker or never having worked, a lower income, having no additional health insurance, a low self-perception of HIV risk, not knowing any PLWHA, a high stigma score towards PLWHA, perceiving oneself as heterosexual or not answered, having had no or only one sexual partner during the previous 5 years, not being in a couple relationship or living in a couple relationship at the time of the survey, having had no or only one couple relationship in one’s lifetime, not having a history of STIs, living alone, and the sense of belonging to a community. All the contextual variables tested were associated with the outcome. On the other hand, having a regular general practitioner, having social support and practicing a religion were not associated with no lifetime HIV testing in the men.

In multivariate multilevel analysis (Table 2), an area-level effect was found in the null model (level 2 variance of 0.2898 [0.1087], *p* = 0.008). Model 1 (with demographic and socioeconomic variables) showed that the factors associated with no lifetime HIV testing were all the age groups, except 30–44 years, a low or an intermediate education level, never having worked, and health insurance status (having health insurance specifically for low-income individuals was associated with a lower likelihood of never having been tested than being insured through the usual system, with or without additional insurance). Introducing these characteristics reduced the level 2 variance by 14 %. In Model 2, the factors associated with our outcome included a low self-perception of HIV risk, a high stigma score towards PLWHA, not knowing any PLWHA, having had no or only one lifetime couple relationship, and not having a history of STIs. Introducing these characteristics led to a 53 % reduction in the initial level 2 variance. When further introducing neighborhood characteristics, only the proportion of immigrants in the neighborhood of residence was significant. In Model 3, the area-level variance (0.0978 [0.0803]) was no more significantly different from zero when both the individual and area-level variables were taken into account, and the MOR gradually decreased from 1.67 in the empty model to 1.35 in the full model.

**Table 2:** Factors associated with never-testing for HIV among men (*n* = 1261) as determined by multilevel multivariate logistic regression

		Model 1	Model 2	Model 3
		aOR [95 % CI]	aOR [95 % CI]	aOR [95 % CI]
Individual variables				
Age	18–29 years	2.07 [1.32–3.24]	1.88 [1.11–3.24]	1.86 [1.08–3.18]
	30–44 years	Ref.	Ref.	Ref.
	45–59 years	1.38 [0.97–1.96]	1.76 [1.20–2.58]	1.77 [1.21–2.59]
	60–69 years	2.89 [1.88–4.42]	3.16 [1.98–5.04]	3.23 [2.02–5.14]
Education level				
	None or primary school	3.29 [1.83–5.94]	2.82 [1.49–5.33]	2.51 [1.32–4.78]
	Secondary school	1.77 [1.24–2.51]	1.72 [1.18–2.52]	1.63 [1.11–2.39]
	Postsecondary	Ref.	Ref.	Ref.
Socio-occupational category				
	Never worked	3. 33 [1.42–7.76]	3.62 [1.44–9.09]	3.34 [1.34–8.33]
	Blue collar	1.24 [0.73–2.09]	1.06 [0.60–1.89]	0.99 [0.56–1.77]
	Lower white-collar	0.81 [0.52–1.24]	0.74 [0.46–1.18]	069 [0.43–1.10]
	Tradespeople, etc.	0.91 [0.59–1.40]	0.95 [0.60–1.52]	0.90 [0.56–1.44]
	Upper white-collar	Ref.	Ref.	Ref.
Health insurance status				
	HI + additional insurance	2.30 [1.29–4.09]	2.23 [1.19–4.18]	2.34 [1.24–4.39]
	HI for the poor	Ref.	Ref.	Ref.
	HI with no additional insurance	2.43 [1.28–4.63]	2.43 [1.21–4.87]	2.46 [1.22–4.94]
Self-perception of HIV risk				
	High		Ref.	Ref.
	Low		3.41 [2.29–5.08]	3.33 [2.24–4.96]
Knowing PLWHA				
	Yes		ref	ref
	No		1.54 [1.09–2.18]	1.57 [1.12–2.22]
Stigma towards PLWHA				
	Score ≤ 2		Ref.	Ref.
	Score > 2		1.70 [1.23–2.34]	1.67 [1.21–2.30]
Lifetime number of couple relationships				
	None		2.96 [1.75–5.00]	2.97 [1.75–5.01]
	One		2.19 [1.53–3.14]	2.16 [1.51–3.10]
	≥ 2		Ref.	Ref.
History of STI				
	Yes		Ref.	Ref.
	No		2.88 [1.64–5.07]	2.89 [1.65–5.06]
Neighborhood variables				
Proportion of immigrants				
	First quartile			1.04 [0.64–1.70]
	Second quartile			ref
	Third quartile			1.12 [0.68–1.85]
	Fourth quartile			1.98 [1.18–3.33]
Level 2 variance		0.2495 (0.1064)	0.1545 (0.0942)	0.0978 (0.0803)
*p*-value		0.019	0.101	0.223
MOR		1.61	1.45	1.35

Empty model: level 2 variance = 0.2898 (0.1087); *p* = 0.008; MOR = 1.67

### Factors associated with no lifetime HIV testing in women

In the women (Table 1), the factors positively associated with never-testing for HIV in univariate analysis were a younger (< 29 years) or an older age (> 60 years), being a foreigner or a French person born to at least one foreign parent, not having a child < 20 years of age, a low education level, not being actively employed, being a blue–collar worker or never having worked, a lower income, not having additional health insurance or having insurance for low-income individuals, a low self-perception of HIV risk, not knowing any PLWHA, a high stigma score towards PLWHA, being self-reported as heterosexual or not answered, having had no or only one sexual partner during the previous 5 years, not being in a couple relationship at the time of the survey, never having been in a couple relationship, not having a history of STIs, weak social support, a sense of belonging to a community, and practicing a religion on a regular basis. On the other hand, neither having a regular practitioner nor living alone was associated with never having been tested for HIV. All the contextual variables were significantly associated with the outcome.

In multivariate multilevel analysis (Table 3), an area-level effect was found in the null model (area-level variance of 0.1864 [0.0698], *p* = 0.008). The following characteristics were selected in Model 1: the age groups other than 30–44 years, being a foreigner, not having a child < 20 years of age, a low or intermediate education level, not being actively employed, never having worked or being in an intermediate socio-occupational category, and not having additional health insurance. Introducing these characteristics reduced the level 2 variance by 38 %, and the area-level variance (0.1179 [0.0637]) was no more significantly different from zero. In Model 2, the attitudes and behavioral factors associated with never having been tested for HIV included a low self-perception of HIV risk, not knowing any PLWHA, a high stigma score towards PLWHA, having had no or only one sexual partner during the previous 5 years, not being in a couple relationship or being in a cohabiting couple relationship at the time of the survey, never having been in a couple relationship, not having a history of STIs, and a sense of belonging to a community. In the full model, the only neighborhood variable selected in the absence of any individual covariables (the neighborhood proportion of inhabitants with a low education level) was not significantly associated with the outcome. Overall, the successive adjustments had smaller impacts on level 2 variance and MOR in the woman than in the men.

**Table 3:** Factors associated with never-testing for HIV among women (*n* = 1360) as determined by multilevel multivariate logistic regression

		Model 1	Model 2
		aOR [95 % CI]	aOR [95 % CI]
Individual variables			
Age	18–29 years	1.70 [1.12–2.56]	1.66 [1.04–2.66]
	30–44 years	Ref.	Ref.
	45–59 years	2.15 [1.54–2.99]	2.18 [1.50–3.17]
	60–69 years	2.55 [1.60–4.05]	2.69 [1.57–4.62]
Immigration status			
	French/French parents	Ref.	Ref.
	French/foreign parent (s)	1.25 [0.91–1.73]	1.07 [0.75–1.55]
	Foreigner	1.55 [1.06–2.27]	1.37 [0.89–2.10]
Child under 20 years of age			
	Yes	Ref.	Ref.
	No	2.94 [2.17–3.99]	3.49 [2.41–5.07]
Education level			
	None or primary school	3.04 [1.77–5.23]	2.61 [1.45–4.71]
	Secondary school	1.57 [1.15–2.12]	1.52 [1.08–2.13]
	Post-secondary	Ref.	Ref.
Employment status			
	Actively employed	Ref.	Ref.
	All others	1.54 [1.12–2.12]	1.11 [0.78–158]
Socio-occupational category			
	Never worked	2.39 [1.39–4.10]	1.81 [0.99–3.27]
	Blue collar	1.28 [0.59–2.80]	1.02 [0.44–2.36]
	Lower white-collar	1.30 [0.88–1.91]	1.12 [0.74–1.72]
	Tradespeople, etc.	1.71 [1.15–2.54]	1.82 [1.18–2.80]
	Upper white-collar	Ref.	Ref.
Health insurance status			
HI + additional insurance	Ref.	Ref.	
	HI for the poor	0.95 [0.58–1.54]	1.29 [0.75–2.22]
HI with no additional insurance	1.72 [1.15–2.57]	2.09 [1.32–3.32]	
Self-perception of HIV risk			
	High		Ref.
	Low		5.04 [3.43–7.41]
Knowing PLWHA			
	Yes		Ref.
	No		2.01 [1.49–2.71]
Sexual partnerships during previous 5 years			
	No partnerships		2.15 [1.26–3.67]
	One partnership		1.73 [1.12–2.68]
	Multiple partnerships		Ref.
Couple status at the time of the survey			
	No relationship		2.35 [1.15–4.78]
	Love affair		2.03 [0.97–4.27]
	Non-cohabiting couple		Ref.
	Cohabiting couple		3.25 [1.57–6.72]
Lifetime number of couple relationships			
	None		2.63 [1.55–4.46]
	One		1.39 [0.99–1.93]
	≥ 2		Ref.
History of STI			
	No		1.62 [1.05–2.50]
	Yes		Ref.
Sense of belonging to a community			
	Yes		1.33 [0.99–1.79]
	No		Ref.
Level 2 variance		0.1179 (0.0637)	0.1655 (0.0804)
*P*-value		0.064	0.039
MOR		1.39	1.47

Empty model: level 2 variance = 0.1864 (0.0698); *p* = 0.008; MOR = 1.51

## Discussion

In addition to the socioeconomic factors usually observed to be associated with prevention attitudes and practices, we found that the absence of any lifetime HIV testing was associated with a low risk perception, health insurance status, not knowing any PLWHA, a high stigma score towards PLWHA, a low lifetime number of couple relationships, the absence of any history of STIs and, for men only, the neighborhood proportion of immigrants.

Our sample was limited to the Paris metropolitan area, which limits its external validity, particularly in nonurban contexts. However, since this region bears the largest part of the HIV burden in mainland France, our conclusions could be useful in helping manage the epidemic [26]. Unfortunately, because of the sampling design, we were unable to reach the most vulnerable populations, such as homeless people (among whom immigrants are overrepresented) [27]. Also, we interviewed only French-speaking individuals, thereby excluding still more foreigners but non-French-speaking individuals accounted for 5 % of the initial sample. If they had been included in the SIRS cohort, the difference between French persons and foreigners may have been accentuated by the inclusion of more individuals not reached by prevention messages.

The participants were interviewed retrospectively about any previous HIV testing. The last test had been performed on average about 5 years before the survey date. Thus, there was a possible recall bias concerning the circumstances of the last test. In addition, some respondents may have confused certain routine laboratory tests with the HIV screening test, which would have led to an overestimation of HIV testing, especially among recent immigrants and less educated and/or less health-literate individuals.

Many studies on the utilization of health-care services have shown that people with a low SES are less likely to avail themselves of preventive care, even in countries where national health services and/or universal health insurance should preclude any financial obstacles to such care [28]. For instance, we reported similar socioeconomic gradients in women’s cancer screening in the same survey population [29, 30].

Taking into account certain indicators of sexual behaviors and attitudes toward PLWHA enabled us to explore the contribution of these factors to some but not all of the observed socioeconomic and demographic differences. In particular, due to a lack of statistical power, we were unable to find a significant association with (self-reported) sexual orientation, since the number of participants who indicated that they were bi- or homosexual was too small in both genders (58 men and 16 women). Moreover, these numbers were probably underestimated, particularly in certain immigrant populations where sexuality and especially homosexuality are still taboo and/or punished by law in their country of origin [31].

The fact that younger people of both sexes were less likely to have been tested for HIV is particularly worrisome. Of course, HIV prevalence in young heterosexual people remains low, with the result that they are not the most at risk for HIV exposure (until now). However, a recent KABP study in the French capital region found that people in this age group had a poorer knowledge and a weaker perception of HIV risk compared not only to the other age groups in 2010, but also to the same age group interviewed previously, between 1992 and 2004 [26]. Indeed, in our study, 70.5 % of the men and 34.2 % of the women in this age group reported that they had had more than one sexual partner during the previous 5 years (compared to 24.9 % and 14.5 %, respectively, of the rest of the population), while in a regional KABP study, only 39 % of the men and 27 % of the women in this age group indicated that they had used a condom at last intercourse (compared to 45 % and 36 % in 2004, respectively) [26]. We also observed, in a previous analysis, that of the 655 HIV-negative individuals aged 18 to 29 years who had answered the question about their intention regarding protection in 2010, 376 (57.4 %) reported that they consistently used condoms to protect themselves from HIV (men more often than women: 71.0 % vs. 43.3 %, respectively, *p* < 0.001) [32].

We also observed that older age was independently associated with never-testing for HIV in both genders. The recent ANRS-Vespa2 study found that older people were also at higher risk for late presentation of HIV in all the subpopulations that were specifically examined, e.g., heterosexual male and female immigrants born in sub-Saharan Africa or North Africa and North Africans who practice a religion. Moreover, MSM over 50 who did not define themselves as gay were at higher risk for late HIV diagnosis [33]. Such a lower level of HIV testing in older individuals could certainly have major consequences on the epidemiology of HIV infection.

In a previous analysis of the same cohort in 2005 (with far fewer individual characteristics obtained by interview and analyzed), we found that gender, socioeconomic status, immigration status and neighborhood of residence were barriers to HIV testing in the adult population in the Paris metropolitan area [17]. We also found that measures aimed at increasing HIV testing in sub-Saharan immigrants may have been effective, given that people from sub-Saharan Africa were more likely to have been tested in their lifetime (78.5 %) than those of French (56.2 %) or Maghreb (39.7 %) origin (*p* < 0.0001) [34]. In 2010, we found that women with a sense of belonging to a community and, to a lesser extent, foreign women were less likely to have been HIV-tested than other women. Actually, these two characteristics are obviously correlated, given that a sense of belonging to a community is shared by 49 % of foreign women and 45 % of French women born to at least one foreign parent (compared to 22 % of French women born to French parents, *p* < 0.0001). In men, these figures were, respectively, 49 %, 45 % and 22 % (*p* < 0.0001).

In our study, we found that individuals with a low lifetime number of couple relationships were less likely to have been tested. Another French study among late-tested HIV-positive individuals found that both male and female heterosexuals in a steady relationship and with children felt that they were less at risk for HIV infection and that this put them at greater risk for never having been tested in the absence of symptoms [35]. Generally speaking, since they are not priority targets for testing promotion, individuals at low risk for HIV infection are at high risk for not being tested for HIV and for late diagnosis [36]. In our study, a low self-perceived risk for HIV infection was associated with no HIV testing in both sexes. Some studies have reported similar observations in immigrants in Portugal [6] and Spain [7], and in pregnant women in other European countries [2, 37, 38]. Deblonde et al., in a systematic review in Europe, found that the main barrier to HIV testing are a low risk perception, fear of HIV disease and of HIV diagnosis disclosure, and lack of access to health services. In our study. the two responses (fear of disease and fear of disclosure) were given, respectively, by only 3 % and 2 % of the never-testers. A minor gradient was observed for fear of disclosure according to immigration status in both sexes (respectively, 0 %, 0.5 % and 8.4 % in the women and 1 %, 1.8 % and 2.7 % in the men).

The men with a high stigma score towards PLWHA and those who did not know any PLWHA among family, friends or coworkers were more likely to be never-testers. As suggested by Burkholder et al. [39], since they do not empathize or identify with PLWHA, people who stigmatize them cannot perceive themselves as possibly being concerned by this matter or at risk for HIV infection. In other words, people with stigmatizing attitudes may perceive a greater distance between themselves and those with the disease [31]. Stigma serves to emphasize and enhance the differences between the stigmatizers and those being stigmatized [40].

The association observed between no HIV testing and no history of STIs could be due to the fact that French health authorities recommend proposing an HIV test whenever an STI is suspected [41]. Practically, this recommendation seems to be followed by some health professionals but not a majority of them. Indeed, in a French study in newly diagnosed HIV-infected people carried out in 2010, only half of the individuals with STIs during the 3 years prior to HIV diagnosis had actually received an HIV testing proposal from a health-care provider [42] .

Lastly, our study found a contextual effect on no lifetime HIV testing in men. Indeed, the decrease in the level 2 variance and its *p*-values showed that introducing the proportion of immigrants living in the neighborhood of residence explained part of the observed differences between neighborhoods. In contrast, in women, the significant differences in the observed crude prevalence rates of HIV testing between neighborhoods were mainly explained by a composition effect (i.e., by individual characteristics).

The men living in neighborhoods with a high proportion of immigrants (more than 28 %) were two times less likely to have been HIV-tested those living in neighborhoods with a proportion of immigrants between 17 and 23 %. This could be explained by a number of norms shared by immigrant men in these neighborhoods, such as the fear of stigmatization or discrimination related to being tested for HIV (regardless of the result). Indeed, we observed that foreigners and French persons born to at least one foreign parent were more likely to have a positive stigma score than French persons born to French parents (61.0 %, 48.2 % and 34.3 %, respectively, in men, *p* < 0.0001; 60.2 %, 48.4 % and 31.3 %, respectively, in women, *p* < 0.0001). Similarly, foreigners and French persons born to at least one foreign parent were less likely to report that they knew a PLWHA than French persons born to French parents, (19.2 %, 28.8 % and 39.0 %, respectively, in men, *p* < 0.0001; 28.7 %, 27.6 % and 42.2 %, respectively, in women, *p* < 0.0001). Lastly, not knowing any PLWHA was significantly associated with a positive stigma score: 47.6 % of the men who did not know any PLWHA had a positive stigma score versus 28.1 % of those who did (*p* <0.001). In the women, these figures were, respectively, 47.9 % and 23.6 % (*p* <0.001).

No lifetime HIV testing could also be a consequence of differentiated practices by health professionals according to their patients’ social or demographic characteristics. For instance, in 2003, a French study found that the factors associated with the lack of proposing HIV/AIDS and hepatitis B and C screening in general practice to underprivileged immigrants were gender (women were screened less for HBV and HCV infection) and being from a non-sub-Saharan African country (especially from North Africa) for all three viruses [43].

## Conclusions

At a time when, in France, many people remain unaware of their HIV status, when the proportion of people screened late is not decreasing and when many practitioners are not adhering to the recommendations for universal screening in primary care [44], it seems more important than ever that HIV screening services target not only the high-risk populations, but also those with a lower self-perceived risk of infection and/or at high risk for stigma, such as younger and older people, those in a steady relationship, and men living in immigrant neighborhoods. In 2016, a new French policy will merge (previously split) HIV, viral hepatitis and/or STI screening centers into comprehensive sexual health centers, which will provide all these screening tests, as well as information, contraception, STI care and linkage to specialist care. This will possibly increase access to HIV screening for those who stay away from HIV testing services. Also, community-based and/or outreach, combined, rapid screening test services should not be limited in France to MSM communities but rather extended to other communities, in particular, immigrant communities other than just that from sub-Saharan Africa.
